# Frailty and the psychosocial components of the edmonton frail scale are most associated with patient experience in older kidney transplant candidates – a secondary analysis within the kidney transplantation in older people (KTOP) study

**DOI:** 10.3389/fneph.2022.1058765

**Published:** 2023-01-17

**Authors:** Amarpreet K. Thind, Shuli Levy, David Wellsted, Michelle Willicombe, Edwina A. Brown

**Affiliations:** ^1^ Centre for Inflammatory Disease, Department of Immunology and Inflammation, Imperial College London, London, United Kingdom; ^2^ Hammersmith Hospital, Imperial College Healthcare NHS Trust, London, United Kingdom; ^3^ The Centre for Health Services and Clinical Research, The University of Hertfordshire, Hertfordshire, United Kingdom

**Keywords:** kidney transplantation, older people, frailty, quality of life, patient experience

## Abstract

**Background:**

Older people with end-stage kidney disease (ESKD) are vulnerable to frailty, which impacts on clinical and experiential outcomes. With kidney transplantation in older people increasing, a better understanding of patient experiences is necessary for guiding decision making. The Kidney Transplantation in Older People (KTOP):impact of frailty on outcomes study aims to explore this. We present a secondary analysis of the Edmonton Frail Scale (EFS) and its relationship with patient experience scores.

**Methods:**

The KTOP study is a single centre, prospective study, which began in October 2019. All ESKD patients aged ≥60 considered for transplantation at Imperial College Renal and Transplant Centre were eligible. Frailty was assessed using the EFS and 5 questionnaires assessed patient experience and quality of life (QoL) (Short Form-12(v2), Palliative Care Outcome Scale–Symptoms Renal, Depression Patient Health Questionnaire-9, Illness Intrusiveness Ratings Scale, Renal Treatment Satisfaction Questionnaire). The EFS was divided into 4 subdomains (psychosocial, physical function, medical status, and general health) and then compared with the questionnaire scores.

**Results:**

210 patients have been recruited (aged 60-78), 186 of whom completed EFS assessments. 118 (63.4%) participants were not frail, 36 (19.4%) vulnerable, and 32 (17.2%) were frail. Worse frailty scores were associated with poorer patient experience and QoL scores across all questionnaires. Severe deficits in the EFS psychosocial subdomain showed a statistically significant association with higher depression screen scores (coefficient 4.9, 95% CI 3.22 to 6.59), lower physical (coefficient -4.35, 95% CI -7.59 to -1.12) and mental function scores (coefficient -8.33, 95% CI -11.77 to -4.88) from the Short Form-12(v2), and lower renal treatment satisfaction scores (coefficient -5.54, 95% CI -10.70 to -0.37). Deficits in the physical function and medical status EFS subdomians showed some association with patient experience scores.

**Conclusion:**

In the KTOP study cohort at recruitment vulnerable and frail candidates reported worse QoL and patient experiences. Severe deficits in the psychosocial subdomains of the EFS showed a strong association with patient experience and QoL, whilst physical function and medical status deficits showed a lesser association. This has highlighted specific EFS domains that may be suitable for targeted interventions to improve experiences and optimise outcomes.

## Introduction

The end stage kidney disease (ESKD) population is ageing, with older people (aged >65) representing the age group with the highest incidence of ESKD (1, 2). Across all age groups ESKD populations experience higher rates of frailty compared with the general population (3, 4). Frailty represents a ‘state of increased vulnerability to physical stressors as a result of progressive and sustained degeneration in multiple physiological systems’, resulting in a spectrum of deficits which confer an increased risk of adverse outcomes (5, 6). With age, comorbidity, and prolonged exposure to kidney disease all recognised as contributing to and even accelerating the development of frailty, older people with ESKD are particularly vulnerable to frailty and enduring the associated consequences (5, 7, 8).

Having ESKD has been shown to considerably impact on quality of life (QoL) at all ages, however older people receiving dialysis report lower QoL scores compared with younger adults (9, 10). Similarly, older ESKD patients put forward for kidney transplantation report a decline in QoL scores whilst on the waitlist, followed by an improvement in QoL scores following a transplant to a level which equals that in the age-matched general population (11, 12). Invariably the experiences of older kidney transplant candidates and the degree to which QoL may improve following a transplant is highly dependent on the candidate’s ability to withstand the stressors of dialysis, navigate the waitlist, and then successfully undergo transplantation, with each stage likely to be affected by the presence of frailty (3).

Frailty is independently associated with worse health related QoL scores throughout the stages of chronic kidney disease (CKD) and into ESKD, with frail transplant candidates reporting lower QoL scores at the time of transplantation, compared with non-frail candidates (9, 13, 14). However frail candidates also experience the greatest improvement in QoL across multiple domains following a transplant, suggesting they stand to gain the most (9, 13, 14). In an age group where increases in life expectancy from transplantation may be limited, understanding and optimising QoL and the experiences of older people with ESKD, is of paramount importance and should be the driving force behind decision making for this cohort (15, 16).

With the impact of frailty on clinical outcomes and QoL in patients with ESKD well established, current research has shifted towards exploring strategies to address and manage frailty (2, 9, 17, 18). Integral to this is understanding the specific components of frailty which may be impacting on patient experiences. This would help identify areas that are suitable for targeted intervention, and to aid patient specific discussions around risk assessment and treatment options. The aims of this study were therefore to (1) describe how QoL and patient reported experience in older kidney transplant candidates varies by frailty status, and (ii) investigate the relationship between components of the Edmonton Frail Scale (EFS) and patient experience scores. This work is a secondary analysis within the wider Kidney Transplantation in Older People (KTOP): impact of frailty on outcomes study (19).

## Methods and materials

The KTOP study is a single centre, mixed methods, observational study being conducted at the Imperial College Renal and Transplant Centre in West London, UK. The KTOP study consists of a questionnaire study (KTOP: impact of frailty on outcomes) and a qualitative study (KTOP: understanding the patient’s experience). The overall aim of KTOP is to achieve a better understanding of the experiences of older people with ESKD, as they wait for and undergo kidney transplantation, with a specific focus on frailty, cognition, QoL and patient experiences, and clinical outcomes. A full description of the KTOP study protocol has been previously described (19). This manuscript represents a secondary analysis from the questionnaire study describing the results from the cohort at recruitment.

The KTOP: impact of frailty on outcomes questionnaire study began in October 2019 and will continue until June 2023. Favorable ethical approval for the study was received from Yorkshire and the Humber Leeds West Research Ethics Committee and Health Research Authority (REC reference 19/YH/0287).

All patients aged ≥ 60 years old, being worked up for kidney transplantation (living or deceased donor) or ‘active’ on the national kidney transplant waitlist, were eligible for recruitment into the study. Patients with substantial language barriers were excluded from the study. Written informed consent was obtained from all recruited participants.

Following recruitment into the study, participants completed a set of baseline questionnaires, including an assessment of frailty using the EFS, and 5 questionnaires to evaluate patient experience and QoL. These questionnaires included: the (1) Short Form-12 version 2 (SF-12(v2)) as a global assessment of QoL, providing physical function and mental function summary scores, (2) Depression Patient Health Questionnaire–9 (PHQ-9) a screen for depressive symptoms, (3) the Palliative Care Outcome Scale – Symptoms Renal (POS-S Renal) measuring symptom burden, (4) Illness Intrusiveness Ratings Scale (IIRS) and (5) Renal Treatment Satisfaction Questionnaire (RTSQ), both evaluating the impact of ESKD and kidney replacement therapy on participants lives. Each of these questionnaires has been validated for use in people with chronic diseases and were chosen based on recommendations made by the multi-disciplinary KTOP study investigator team (19).

The EFS was chosen as it is a well validated, reliable, and easy to perform frailty tool, which gives both an overall assessment of frailty status (total score) and assesses individual components of frailty (20). **Table 1** summarises the EFS, including the components assessed by the EFS, the scoring for each component, which is based on the presence or absence of the component or the severity of the deficit present. A total score is calculated, and this score then corresponds to a frailty status. An EFS total score of ≤5 translates to the participants being ‘not frail’, a score of 6-7 represents being ‘vulnerable’ to frailty, and a score ≥8 represents being ‘frail’.

**Table 1:** Summary of the edmonton frail scale.

Frailty Domain	Item	Score	Subdomain Created
**Functional Independence**	Number of activities of daily living that require help	0 – 0 or 1 activities 1 – 2 to 4 activities 2 – 5 to 8 activities	**Physical Function**
**Functional performance**	Time up and go assessment	0 – 0 to 10 seconds 1 – 11 to 20 seconds 2 - > 20 seconds	
**Cognition**	Clock draw with specified time	0 – No errors 1 - Minor spacing errors 2 - Other errors	**Psychosocial**
**Social support**	Availability of help when required	0 – Always 1 – Sometimes 2 - Never	
**Medication adherence**	Forget to take medications	0 – No 1 – Yes	
**Mood**	Often feel sad or depressed	0 – No 1 – Yes	
**Hospital admissions**	Hospital admissions in last year	0 – No admissions 1 – 1 to 2 admissions 2 - >2 admissions	**Medical Status**
**Medication use**	Taking ≥ 5 medications daily	0 – No 1 – Yes	
**Nutrition**	Noticeable weight loss	0 – No 1 – Yes	
**Continence**	Loss of urinary control	0 – No 1 – Yes	
**General Health Status**	Subjective description of health	0 – Excellent, very good, good 1 – Fair 2 – Poor	**General Health Status**

Table adapted from the Edmonton Frail scale first developed and described by Rolfson et al. (2006) (20). The original EFS components were combined to create the following subdomians; physical function - functional independence and functional performance combined, psychosocial - cognition, social support, medication adherence and mood combined, medical status - hospital admissions, medication use, nutrition and continence combined. The General Health Status domain was retained as a seperate subdomian.

For this secondary analysis subdomains within the EFS were created by combining EFS components that assess similar aspects of a participant’s life (**Table 1**). The following categories were created; (1) physical function - functional independence and functional performance combined, (2) psychosocial - cognition, social support, medication adherence, and mood combined, (3) medical status - hospital admissions, medication number, nutrition, and continence combined. The ‘general health status’ (4) component of the EFS was retained as an individual variable, as this question represents a broad screen of the participants self-perceived health rather than a specific factor that contributes to a wider domain within the EFS. These subdomains were created using both a theoretical approach and a data driven approach, where a correlation table was used to determine the degree of association between each of the EFS components. For each of the 4 identified EFS subdomains, the total score for the subdomian was calculated and the patients were organised into 3 groups based on tertial scores, representing those with the lowest subdomain score (group 1 – little to no deficits) through to the highest subdomain score (group 3 – most severe deficits). The subdomain scores calculated were not validated using scores from related scales as the focus of this work was to present and describe a new pragmatic approach to using the EFS and apply this approach to the KTOP study cohort. This method is novel and a separate date set will be required to assess its validity, which should be conducted as a separate piece of work. Together with the questionnaire responses demographic and medical history data was also collected. The data presented in this manuscript represent the results of the EFS and patient experience questionnaires completed by KTOP participants at recruitment and includes only those participants recruited prior to receiving a kidney transplant.

The demographic and clinical characteristics of the study cohort were analysed using descriptive statistics. Linear regression was used to determine the relationships between frailty status (from total EFS score) and the patient experience scores, and the association between the frailty subdomains and the patient experience scores. All analyses were adjusted for gender, age, and comorbidity burden (determined by the Charlson Co-morbidity index). The level of statistical significance was set at p <0.05. All analyses were completed using Stata/BE version 17.0 (StataCorp LLC, Texas), with supervision from the University of Hertfordshire (Centre for Health Services and Clinical Research).

The wider KTOP study remains active and will continue until 2023. Subsequent publications from the KTOP study investigator group will present the broader results of the study on completion.

## Results

Two hundred and ten patients have been recruited into the KTOP study since October 2019. Pre-transplant EFS assessments were available for 186 participants. Based on these assessments the mean EFS score for the cohort was 4.94 (SD 2.55, 95% CI 4.57 – 5.30), with 118 participants (63.4%) identified as not frail (EFS total score 0-5), 36 participants (19.4%) identified as vulnerable (EFS total score 6-7), and 32 participants (17.2%) identified as frail (EFS total score ≥8). **Table 2** summarises the demographics for the study cohort arranged by frailty status. Completion of the patient experience questionnaires varied across the participants, resulting in 184 completed SF-12 (v2) questionnaires, 181 completed PHQ-9 and POS-S Renal questionnaires, 180 completed RTSQs and 179 completed IIRS questionnaires. The variability in questionnaire responses resulted from some participants declining to complete certain questionnaires (e.g. PHQ-9 depression screen) and/or participants finding the volume of questionnaires and the time required to complete them troublesome, therefore they did not finish completing all questionnaires as planned.

**Table 2:** Clinical demographics of study cohort by frailty status.

Characteristic		Not frail (n=118)	Vulnerable (n=36)	Frail (n=32)
**Age (mean, range) (years)**		66 (60-77)	65.1 (60-78)	64.6 (60-74)
**Gender**	**Male**	78 (66.1)	26 (72.2)	17 (53.1)
**Ethnicity**	**South Asian**	57 (48.3)	14 (38.9)	15 (46.8)
	**Caucasian**	38 (32.2)	7 (19.4)	3 (9.4)
	**Afro-Caribbean**	16 (13.6)	8 (22.2)	7 (21.9)
	**Middle Eastern**	2 (1.7)	5 (13.9)	5 (15.6)
	**East Asian**	5 (4.2)	2 (5.6)	2 (6.3)
**Cause of ESKD**	**Diabetic nephropathy**	49 (41.5)	16 (44.3)	19 (59.3)
	**Unknown**	13 (11)	8 (22.2)	2 (6.3)
	**Glomerulonephritis**	15 (12.7)	4 (11.1)	4 (12.4)
	**PKD**	13 (11)	1 (2.8)	2 (6.3)
	**Urological**	7 (5.9)	2 (5.6)	1 (3.1)
	**Other**	9 (7.6)	1 (2.8)	0
	**Renovascular disease**	5 (4.3)	2 (5.6)	2 (6.3)
	**Hypertension**	5 (4.3)	1 (2.8)	0
	**FSGS**	2 (1.7)	1 (2.8)	2 (6.3)
**Modality of KRT**	**ICHD**	91 (77.1)	32 (88.9)	30 (93.8)
	**Home-HD**	1 (0.9)	0	0
	**PD**	19 (16.1)	4 (11.1)	1 (3.1)
	**AKCC**	7 (5.9)	0	1 (3.1)
**Vintage (mean, LQ-UQ) (days)**		918 (197-1214)	1073 (475-1450)	1164 (465-1410)
**Previously transplanted**		21 (17.8)	9 (25)	6 (18.8)
**Charlson co-morbidity index score (mean, LQ-UQ)**		5.8 (4-7)	6 (5-7)	6.7 (6-8)

Results presented as n (%) unless otherwise stated. ESKD, end stage kidney disease; KRT, kidney replacement therapy; ICHD, in-centre haemodialysis; HD, haemodialysis; PD, peritoneal dialysis; AKCC, advanced kidney care clinic; LQ, lower quartile; UQ, upper quartile.

### Frailty status and reported Qol scores

**Figure 1:**
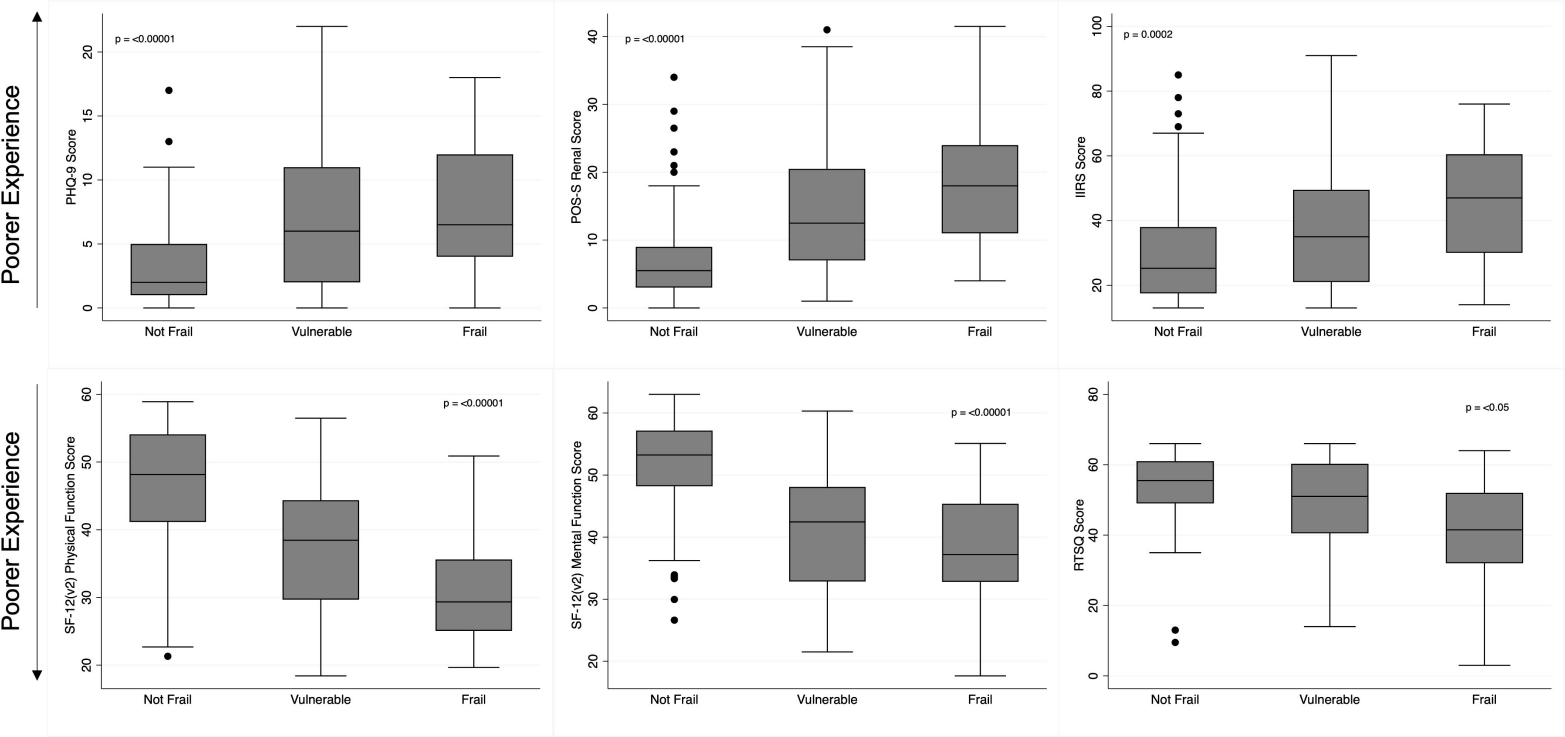
Patient Reported Experience Scores by Frailty Status. Box and whisker plot illustrating the distribution of patient experience questionnaire scores by identified frailty status. PHQ-9, Depression Patient Health Questionnaire, 9; POS-S Renal, Palliative Care Outcome Scale - Symptoms Renal; IIRS, Illness Intrusiveness Ratings Scale; SF-12 (v2), Short-Form- 12 (version 2); RTSQ, Renal Treatment Satisfaction Questionnaire.

The mean reported QoL and patient experience scores and how they varied by the identified frailty status is summarised in **Figure 1**. **Figure 1** demonstrates that as the frailty scores worsen, the mean reported scores across all study questionnaires also worsen. Consequently, in all questionnaires the poorest scores were reported by frail participants, with better scores reported in participants identified as vulnerable, and the best scores reported by those identified as not frail (**Figure 1**). **Table 3** presents the correlation coefficients describing the degree to which questionnaire scores changed as frailty status changed, following adjustment for gender, age, and comorbidity burden. The correlation coefficients all demonstrate that as the frailty scores (translated into frailty statuses) worsen, across all questionnaires poorer patient experience scores are reported, and the degree to which the scores change also increases. In all questionnaires the correlation coefficients between the ‘vulnerable and not frail’ groups, and the ‘frail and not frail’ groups reached statistical significance (p value of <0.05).

**Table 3:** Patient experience scores and their variation by identified frailty status.

Frailty Status	PHQ-9(n=181)	POS-S Renal(n=181)	IIRS(n=179)	SF-12 (v2)		RTSQ(n= 180)
				Physical Function Score (n=184)	Mental Function Score (n=184)	
**Not frail**	*Ref*	*Ref*	*Ref*	*Ref*	*Ref*	*Ref*
**Vulnerable**	3.67 ** (2.08 to 5.25)	7.46 ** (4.48 to 10.43)	7.02 * (0.39 to 13.65)	-8.42 ** (-11.61 to -5.23)	-8.72 ** (-11.90 to -5.53)	-4.71* (-9.17 to -0.25)
**Frail**	3.86 ** (2.13 to 5.59)	9.36 ** (6.06 to 12.67)	13.19 ** (5.84 to 20.54)	-13.57 ** (-17.08 to -10.06)	-12.76 ** (-16.27 to -9.26)	12.76 ** (-17.71 to -7.81)
	Scale 0-22	Scale 0-84	Scale 7-91	Scale 0-100		Scale 0-66
	Higher scores reflect a poorer experience			Lower scores reflect a poorer experience		

Results are presented as the correlation coefficients (confidence interval) describing the change in questionnaire score between the frailty statuses when compared to ‘not frail’ as the reference group, adjusted for age, gender and comorbidity burden. *p value = <0.05, ** p value = <0.001, Ref – reference group, PHQ-9 – Depression Patient Health Questionnaire, 9, POS-S Renal – Palliative Care Outcome Scale - Symptoms Renal, IIRS – Illness Intrusiveness Ratings Scale, SF-12 (v2) – Short-Form- 12 (version 2), RTSQ, Renal Treatment Satisfaction Questionnaire.

### Frailty subdomains and reported QoL scores

The results of the linear regression analysis between the 4 identified EFS subdomains and each of the patient experience questionnaire scores are summarised in **Table 4**.

**Table 4:** Patient experience scores and their variation by edmonton frail scale subdomains.

Edmonton Frail Scale Subdomain		PHQ-9(n=181)	POS-S Renal(n=181)	IIRS(n=179)	SF-12 (v2)		RTSQ(n=180)
					Physical Function Score (n=184)	Mental Function Score (n=184)	
**Physical Function**	**Group 1** (n=67)	*Ref*	*Ref*	*Ref*	*Ref*	*Ref*	*Ref*
	**Group 2** (n=86)	-0.34 (-1.67 to 0.98)	2.96 (0.32 to 5.61)*	1.12 (-4.88 to 7.12)	-4.42 (-6.93 to -1.91)*	-3.75 (-6.43 to -1.07)*	0.87 (-3.18 to 4.92)
	**Group 3** (n=33)	-0.32 (-2.12 to 1.48)	3.08 (-0.54 to 6.71)	1.67 (-6.51 to 9.85)	-8.47 (-11.91 to -5.03)**	-3.85 (-7.51 to -0.18)*	-3.47 (-9.02 to 2.08)
**Psychosocial**	**Group 1** (n=106)	*Ref*	*Ref*	*Ref*	*Ref*	*Ref*	*Ref*
	**Group 2** (n=53)	0.92 (-0.42 to 2.25)	0.49 (-2.19 to 5.61)	-0.57 (-6.68 to 5.55)	-1.62 (-4.13 to 0.90)	-1.79 (-4.47 to 0.90)	-3.63 (-7.74 to 0.47)
	**Group 3** (n=27)	4.90 (3.22 to 6.59)**	6.32 (2.95 to 9.69)**	5.22 (-2.39 to 12.84)	-4.35 (-7.59 to -1.12)*	-8.33 (-11.77 to -4.88)**	-5.54 (-10.70 to -0.37)*
**Medical Status**	**Group 1** (n=87)	*Ref*	*Ref*	*Ref*	*Ref*	*Ref*	*Ref*
	**Group 2** (n=59)	-0.30 (-1.64 to 1.04)	0.51 (-2.16 to 3.18)	0.65 (-5.42 to 6.72)	-2.09 (-4.56 to 0.51)	-0.44 (-3.13 to 2.26)	-3.42 (-7.53 to 0.69)
	**Group 3** (n=40)	0.33 (-1.17 to 1.83)	1.53 (-1.47 to 4.53)	7.20 (0.41 to 13.99)*	-3.63 (-6.50 to -0.76)*	-2.96 (-6.02 to 0.10)	-7.06 (-11.63 to -2.47)*
**General Health Status**	**Excellent, very good, or good** (n=115)	*Ref*	*Ref*	*Ref*	*Ref*	*Ref*	*Ref*
	**Fair** (n=46)	3.55 (2.18 to 4.93)**	4.95 (2.19 to 7.71)*	10.02 (3.79 to 16.24)*	-7.61 (-10.24 to -4.99)**	-7.45 (-10.24 - -4.65)**	-5.24 (-9.46 to -1.01)*
	**Poor** (n=25)	3.60 (1.77 to 5.42)**	8.05 (4.41 to 11.70)**	12.67 (4.45 to 20.90)*	-12.45 (-15.95 to -8.95)**	-10.17 (-13.9 to -6.45)**	-6.34 (-11.92 to -0.76)*
		Scale 0-22	Scale 0-84	Scale 7-91	Scale 0-100		Scale 0-66
		Higher scores reflect a poorer reported experience			Lower scores reflect a poorer reported experience	

Results are presented as the correlation coefficients (confidence interval) describing the change in questionnaire score between the 4 identified Edmonton Frail Scale subdomains when compared to the least severe group in that category (the reference group), adjusted for age, gender, and comorbidity burden. The approach to defining the 4 subdomains is described in the methods section. The number of participants in each group is presented, however the number of participants that contributed to the analysis for each questionnaire varied according to the response rate for that questionnaire. *p value = <0.05, ** p value = <0.001, Ref – reference group, PHQ-9 – Depression Patient Health Questionnaire – 9, POS-S Renal – Palliative Care Outcome Scale - Symptoms Renal, IIRS – Illness Intrusiveness Ratings Scale, SF-12 (v2) – Short-Form- 12 (version 2), RTSQ, Renal Treatment Satisfaction Questionnaire.

Self-reported general health status (fair and poor) was statistically significantly associated with poorer reported scores across all questionnaires in this study (**Table 4**). More specifically however, group 3 within the psychosocial subdomain (representing participants with the highest psychosocial deficit scores) showed a statistically significant association with poorer scores in the PHQ-9 (correlation coefficient 4.90, p value <0.0001), POS-S Renal (coefficient 6.32, p value <0.0001), SF-12 (v2) physical function (coefficient -4.35, p value 0.009) and mental function scores (coefficient -8.33, p value <0.0001), and renal treatment satisfaction scores (coefficient -5.54, p value 0.036).

Both mild (group 2) and severe deficits (group 3) within the physical function subdomain, were statistically significantly associated with worsening physical function scores (coefficients -4.42 and -8.47, p value 0.001 and <0.0001 respectively) from the SF-12 (V2), and had a weaker association observed with the SF-12 (v2) mental function scores (coefficients -3.75 and -3.85, p value 0.006 and 0.04 respectively). Participants with mild deficits in the physical function subdomain (group 2) also demonstrated a statistically significant association with worsening POS-S renal scores (coefficient 2.96, p value 0.028). No statistically significant association was observed between the PHQ-9, IIRS, and RTSQ scores and any of the physical function subdomain groups.

Only deficits in the most severe medical status subdomain (group 3), were statistically significantly associated with worse SF-12 (v2) physical function scores (coefficient -3.63, p value 0.014), RTSQ scores (coefficient -7.06, p value 0.003) and IIRS scores (coefficient 7.20, p value 0.038). Milder deficits (group 2) in the psychosocial and medical status subdomains were not statistically significantly associated with reported scores in any of the patient experience questionnaires used.

## Discussion

In the KTOP study cohort, which describes older people with ESKD being considered for kidney transplantation, participants identified as vulnerable or frail reported poorer patient experiences across all study questionnaires, when compared to non-frail participants. Furthermore, as the frailty scores worsen the degree to which patient experiences scores declined also increased. The subdomain of the EFS which was most substantially associated with poorer patient experience was those with the most severe deficits in the psychosocial domain (group 3). This group showed a statistically significant association with poorer reported scores in 5 of the 6 patient experience areas assessed by the study questionnaires, highlighting the major contribution that psychosocial factors have on the broad experiences of older kidney transplant candidates. Moreover, participants with milder deficits (group 2) in the physical function and medical status subdomains of the EFS, also showed a statistically significant association with 3 of the 6 patient experience areas, suggesting that these aspects of frailty may also warrant closer attention and exploration.

These findings are important as they help identify which individuals (vulnerable and frail), and more specifically which areas, should be targeted by purposeful interventions to improve patient experiences and outcomes. Our findings suggest that focus should be directed towards those older people most severely affected by psychosocial deficits, but also interventions on physical function and medical status (e.g. medication adherence) will provide some benefit.

Although a strong association with general health status (fair or poor) and each of the patient experience questionnaire scores was demonstrated in this data, this finding is of lesser clinical importance and application. The general health status question in the EFS provides a participant’s perspective on their global health (see **Table 1**). The very nature of this question is expected to strongly associate with the experiences assessed in subsequent questionnaires, as each of the questionnaires aim to capture aspects of a participant’s overall health. The general health status component of the EFS therefore provides limited additional information on specific areas of a participants care or health that could be targeted by support interventions. The association of the patient experience scores with general health status is a valid but expected finding and may prove more useful when used as a global screening question, rather than a component for directing subsequent interventions towards.

Numerous studies have reported that the presence of frailty is associated with worse or lower health related QoL in both the general population and renal populations (9, 14, 21, 22). The findings presented from this study agree and strengthen this well described relationship, however this study goes further in exploring exactly which components of frailty may be contributing most to patient experience and QoL. Established knowledge in this area is limited. Nixon and colleagues (2020) found that ‘self-perceived exhaustion’ was the only component of the Frailty Phenotype that had a statistically significant effect across all domains of the Short Form-36 QoL assessment (14). Although this prior study included adult CKD and ESKD patients of all ages, to some extent their findings are similar to this work where self-perceived general health status (fair and poor) from the EFS was associated with poorer patient experience scores. More relevant however, is that self-perception is likely highly related to an individual’s psychosocial health, both contributing to it and a consequence of it. Therefore the observation made by Nixon and colleagues related to self-perceived exhaustion, may also be echoed in our observation that deficits in the psychosocial subdomain are most associated with the lower patient experience scores and do have a major contribution to patients’ QoL (14).

This study also found that mild deficits in the physical function EFS subdomain (group 2) were associated with poorer scores reported in the POS-S Renal and SF-12 (v2) questionnaires. As the KTOP study cohort consists of older patients with ESKD who are being considered for transplantation, the cohort represents those older people who are deemed robust enough to be eligible for transplantation. Consequently, patients with severe deficits in physical function are not captured in the KTOP study cohort and this may explain why a more frequent association with patient experience scores in the physical function category was observed in the mild deficit group (group 2, which has 86 participants) rather than those with severe deficits (group 3, 33 participants). The finding that mild physical function deficits are associated with lower physical function and mental function scores from the SF-12 (v2) assessment are supported by the results of the Dialysis Outcomes and Practice Patterns (DOPPS) study (23). This study identified that lower functional status was strongly associated with lower physical component scores and mental component scores measured by the Kidney Disease Quality of Life instrument, and that functional status was a strong predictor of patient reported outcomes (23). It must be remembered however, that the DOPPS study investigated QoL in all dialysis patients and therefore was not specific to transplant candidates as in this KTOP cohort (23).

Based on the findings from this work several recommendations for clinical practice can be inferred. This work strengthens the argument that frailty assessments should routinely be included in the care of older people with ESKD, especially those considering transplantation. Frailty assessments facilitate a holistic assessment of candidates and as demonstrated, are integral to understanding the current experiences of older people. This information is necessary for discussing and determining the most appropriate treatment goals, particularly around substantial interventions such as transplantation. The growing need to address the psychosocial aspects of ESKD patients’ lives has also been highlighted in this work. Renal units must make progress towards this by ensuring patients have access to counsellors, social workers, and well-being support services. Equally healthcare professionals should feel comfortable to address these issues and know how to direct patients to relevant services. For older KT candidates, where frailty assessments do identify deficits, early and continued involvement from multi-disciplinary colleagues should be introduced. Implementing any or all of these recommendations will ensure both patients and their transplant units are better prepared and informed of a candidate’s specific needs prior to transplantation, so that they can be appropriately supported and adjusting to life after transplantation is as smooth as possible

Limitations of this work include that this is experience from a single centre, this a secondary analysis, and a novel approach to using the EFS assessment has been applied. The work presented here is a secondary analysis of patient responses within the wider KTOP study. The KTOP study was originally powered to assess differences in patient experience and QoL between frail and non-frail older transplant candidates, and so was not powered to investigate the relationship between the specific EFS components and the questionnaire scores. Consequently, the associations described here must be interpreted cautiously. The EFS was originally tested and validated for use as a complete questionnaire providing a total score (20). The methods described in this study whereby the EFS is broken down into subdomains is novel and not yet tested. Although an exploratory factor analysis to identify and validate subdomains in the EFS could be considered, the focus of this work was to describe and present the approach used in this study, and not for validation of the method described. A large and more representative sample would be required to perform a factor analysis, however this is not available within the current study. This method of cluster selection is therefore limited and there may be measurement error in the analysis, however the pragmatic and theoretical approach used to generate the EFS subdomains will help improve the wider interpretation and clinical application of the findings described. Navarro-Flores et al. have used an exploratory factor analysis to evaluate the structure of the EFS (24). Their validation was performed in a clinical group different to the profile of participants in the KTOP study, their model had quite a poor fit, and their analysis incorporated the general health status of the EFS which our work suggests should be treated as a separate variable (24). The Navarro-Flores approach has therefore not been applied to this data set, however, does demonstrate that the EFS can be decomposed into subscales. The approach described in this work will be carried through to subsequent analyses from the KTOP study, which will help to further assess and validate this method. Additionally, the statistical approach of using a single regression model to analyse the association between the EFS subdomains and each of the questionnaire scores, adds further accuracy to the results presented. This model ensures all other factors are controlled for and therefore the associations observed are independent and robust where they do exist. Creation of these subdomains has allowed enhanced application of the EFS assessment by identifying areas within the assessment that can be targeted by support and intervention strategies. Novel approaches like this are necessary in order to advance the care and management of frailty in older patients with ESKD once it has been identified (18). It should also be noted that the KTOP study is purposely focused on exploring the experiences of older people awaiting kidney transplantation and so younger people (aged <60) are excluded, despite the increased prevalence of frailty well described in ESKD patients even at younger ages. This is intentional as the KTOP study is focused on the older demographic specifically in order to provide more detailed information on experiences in this cohort, which will help improve discussions, risk assessment, and decision making around transplantation in older people, where often outcomes are mixed and decisions related to listing are difficult.

The interplay between frailty, depression, and self-reported patient experience is complicated, with each of these factors often influencing the presence and experience of the other. Teasing out the individual influences is difficult, however our findings clearly demonstrate the increasing need to recognise and address the psychosocial aspects of ESKD patients’ lives. Work by Battaglia et al. supports this practice, having previously demonstrated the high prevalence of psychosocial syndromes in both ESKD patients awaiting transplantation and in kidney transplant recipients (present in approximately 60% of both studied cohorts) (25, 26). Their work also highlights that these conditions are often subtle, have a large overlap, and are better detected using multiple tools (e.g. Diagnostic Criteria for Psychosomatic Research and International Classification of Diseases 10) in order to truly appreciate the burden in ESKD patients (25, 26). The need to address psychosocial care has recently been highlighted as a priority area by many expert groups (27–29). With kidney transplantation often heralded as a ‘magic bullet’ to ESKD by both patients and healthcare professionals alike, developing a better understanding of the experiences of older transplant candidates during this time is essential for guiding appropriate decision making. Ultimately, the KTOP study will add value to this area by providing a holistic, longitudinal, and detailed description of these experiences, and their impact on clinical outcomes, which will then be used to better inform future practice.

## Conclusion

In the KTOP study cohort at recruitment, vulnerable and frail older transplant candidates report poorer Qol and patient experiences. Severe deficits in the psychosocial subdomains of the EFS show a strong association with patient experience and QoL, and therefore represent an area of older peoples ESKD care that should be targeted to improve experiences and optimise outcomes.

## Data availability statement

The datasets presented in this article are not readily available because the data reported in this article is available from the corresponding author on reasonable request. Requests to access the datasets should be directed to a.thind@imperial.ac.uk.

## Ethics statement

The studies involving human participants were reviewed and approved by Yorkshire and the Humber Leeds West Research Ethics Committee and Health Research Authority (REC reference 19/YH/0287). The patients/participants provided their written informed consent to participate in this study.

## Kidney Transplantation in Older People (KTOP) Study Investigator Group

In addition to the individual authors listed in the authorship of this paper, the KTOP Study Investigator Group includes Annabel Rule, Dawn Goodall, Frank JMF Dor, Nicola Evans, Shone Surendran, David Ospalla, Nicola Thomas, and Lina Johansson.

## Author contributions

AT was responsible for conducting the research, collecting and analysing the data, and writing the manuscript. DW provided statistical support for data analysis and interpretation. SL helped edit the manuscript. The KTOP Study Investigator Group, SL, EB and MW all contributed to the study conception and study design. EB and MW provided additional support to the editing of the manuscript and maintained oversight for the work. All authors contributed to the article and approved the submitted version.

## References

[1] Registry, U.R. UK Renal registry 23rd annual report. (Bristol, UK: UK Kidney Association) (2021).

[2] Lorenz EC Kennedy CC Rule AD LeBrasseur NK Kirkland JL Hickson LJ. Frailty in CKD and transplantation. Kidney Int Rep (2021) 6(9):2270–80. doi: 10.1016/j.ekir.2021.05.025 PMC8418946 34514190

[3] Harhay MN Rao MK Woodside KJ Johansen KL Lentine KL Tullius SG. An overview of frailty in kidney transplantation: Measurement, management and future considerations. Nephrol Dial Transplant (2020) 35(7):1099–112. doi: 10.1093/ndt/gfaa016 PMC7417002 32191296

[4] Chowdhury R Peel NM Krosch M Hubbard RE. Frailty and chronic kidney disease: A systematic review. Arch Gerontol Geriatr (2017) 68:135–42. doi: 10.1016/j.archger.2016.10.007 27810661

[5] Nixon AC Bampouras TM Pendleton N Woywodt A Mitra S Dhaygude A. Frailty and chronic kidney disease: Current evidence and continuing uncertainties. Clin Kidney J (2018) 11(2):236–45. doi: 10.1093/ckj/sfx134 PMC5888002 29644065

[6] Clegg A Young J Iliffe S Rikkert MO Rockwood K. Frailty in elderly people. Lancet (2013) 381(9868):752–62. doi: 10.1016/S0140-6736(12)62167-9 PMC4098658 23395245

[7] Wu PY Chao CT Chan DC Huang JW Hung KY. Contributors, risk associates, and complications of frailty in patients with chronic kidney disease: A scoping review. Ther Adv Chronic Dis (2019) 10. doi: 10.1177/2040622319880382 PMC6778996 31632625

[8] van Loon IN Wouters TR Boereboom FT Bots ML Verhaar MC Hamaker ME. The relevance of geriatric impairments in patients starting dialysis: A systematic review. Clin J Am Soc Nephrol (2016) 11(7):1245–59. doi: 10.2215/CJN.06660615 PMC4934838 27117581

[9] McAdams-DeMarco MA Ying H Olorundare I King EA Desai N Dagher N. Frailty and health-related quality of life in end stage renal disease patients of all ages. J Frailty Aging (2016) 5(3):174–9. doi: 10.14283/jfa.2016.106 PMC6205225 29240319

[10] Hall RK. Prioritizing the quality of life of older adults with kidney disease. Nat Rev Nephrol (2021) 17(3):149–50. doi: 10.1038/s41581-021-00397-4 33473235

[11] Lonning K Heldal K Bernklev T Brunborg C Andersen MH von der Lippe N. Improved health-related quality of life in older kidney recipients 1 year after transplantation. Transplant Direct (2018) 4(4):e351. doi: 10.1097/TXD.0000000000000770 PMC5908461 29707622

[12] Lonning K Midtvedt K Bernklev T Brunborg C Andersen MH von der Lippe N. Changes in health-related quality of life in older candidates waiting for kidney transplantation. Nephrol (Carlton) (2018) 23(10):948–56. doi: 10.1111/nep.13117 28734131

[13] McAdams-DeMarco MA Olorundare IO Ying H Warsame F Haugen CE Hall R. Frailty and postkidney transplant health-related quality of life. Transplantation (2018) 102(2):291–9. doi: 10.1097/TP.0000000000001943 PMC5790611 28885489

[14] Nixon AC Bampouras TM Pendleton N Mitra S Brady ME Dhaygude AP. Frailty is independently associated with worse health-related quality of life in chronic kidney disease: A secondary analysis of the frailty assessment in chronic kidney disease study. Clin Kidney J (2020) 13(1):85–94. doi: 10.1093/ckj/sfz038 32083613 PMC7025341

[15] Knoll GA. Kidney transplantation in the older adult. Am J Kidney Dis (2013) 61(5):790–7. doi: 10.1053/j.ajkd.2012.08.049 23261121

[16] Rao PS Merion RM Ashby VB Port FK Wolfe RA Kayler LK. Renal transplantation in elderly patients older than 70 years of age: Results from the scientific registry of transplant recipients. Transplantation (2007) 83(8):1069–74. doi: 10.1097/01.tp.0000259621.56861.31 17452897

[17] Vleut R Abramowicz D Hellemans R. Frailty: A new comorbidity in kidney transplant candidates? Nephrol Dial Transplant (2020) 35(7):1085–7. doi: 10.1093/ndt/gfaa166 32777078

[18] Mayes J Young HML Blacklock RM Lightfoot CJ Chilcot J Nixon AC. Targeted non-pharmacological interventions for people living with frailty and chronic kidney disease. Kidney Dialysis (2022) 2(2):245–61. doi: 10.3390/kidneydial2020025

[19] Thind AK Rule A Goodall D Levy S Brice S Dor FJMF. Prevalence of frailty and cognitive impairment in older transplant candidates - a preview to the kidney transplantation in older people (KTOP): Impact of frailty on outcomes study. BMC Nephrol (2022) 23(1):283. doi: 10.1186/s12882-022-02900-w 35963988 PMC9375902

[20] Rolfson DB Majumdar SR Tsuyuki RT Tahir A Rockwood K. Validity and reliability of the Edmonton frail scale. Age Ageing (2006) 35(5):526–9. doi: 10.1093/ageing/afl041 PMC5955195 16757522

[21] Kojima G Iliffe S Jivraj S Walters K. Association between frailty and quality of life among community-dwelling older people: A systematic review and meta-analysis. J Epidemiol Community Health (2016) 70(7):716–21. doi: 10.1136/jech-2015-206717 26783304

[22] Mansur HN Colugnati FA Grincenkov FR Bastos MG. Frailty and quality of life: A cross-sectional study of Brazilian patients with pre-dialysis chronic kidney disease. Health Qual Life Outcomes (2014) 12:27. doi: 10.1186/1477-7525-12-27 24580960 PMC4234401

[23] Brown EA Zhao J McCullough K Fuller DS Figueiredo AE Bieber B. Burden of kidney disease, health-related quality of life, and employment among patients receiving peritoneal dialysis and in-center hemodialysis: Findings from the DOPPS program. Am J Kidney Dis (2021) 78(4):489–500.e1. doi: 10.1053/j.ajkd.2021.02.327 33872688

[24] Navarro-Flores E de Bengoa Vallejo RB Losa-Iglesias ME Palomo-Lopez P Calvo-Lobo C Lopez-Lopez D. The reliability, validity, and sensitivity of the Edmonton frail scale (EFS) in older adults with foot disorders. Aging (Albany NY) (2020) 12(24):24623–32. doi: 10.18632/aging.202140 PMC7803512 33349621

[25] Battaglia Y Zerbinati L Martino E Piazza G Massarenti S Storari A. Psychosocial dimensions in hemodialysis patients on kidney transplant waiting list: Preliminary data. Transplantology (2020) 1(2):123–34. doi: 10.3390/transplantology1020012

[26] Battaglia Y Martino E Piazza G Cojocaru E Massarenti S Peron L. Abnormal illness behavior, alexithymia, demoralization, and other clinically relevant psychosocial syndromes in kidney transplant recipients: A comparative study of the diagnostic criteria for psychosomatic research system versus ICD-10 psychiatric nosology. Psychother Psychosom (2018) 87(6):375–6. doi: 10.1159/000490000 30391961

[27] Kidney Care UK, N.P.W.G. Pyschosocial health - a manifesto for action. Kidney Care UK, Alton, UK. (2022) p. 1–27. Available at: https://www.kidneycareuk.org/about-kidney-health/living-kidney-disease/mental-health/manifesto/.

[28] Chadban SJ Ahn C Axelrod DA Foster BJ Kasiske BL Kher V. KDIGO clinical practice guideline on the evaluation and management of candidates for kidney transplantation. Transplantation (2020) 104(4S1 Suppl 1):S11–S103. doi: 10.1097/TP.0000000000003136 32301874

[29] Segall L Nistor I Pascual J Mucsi I Guirado L Higgins R. Criteria for and appropriateness of renal transplantation in elderly patients with end-stage renal disease: A literature review and position statement on behalf of the European renal association-European dialysis and transplant association Descartes working group and European renal best practice. Transplantation (2016) 100(10):e55–65. doi: 10.1097/TP.0000000000001367 27472096

